# Confirmation Using Triple Quadrupole and High-Resolution Mass Spectrometry of a Fatal Canine Neurotoxicosis following Exposure to Anatoxins at an Inland Reservoir

**DOI:** 10.3390/toxins14110804

**Published:** 2022-11-18

**Authors:** Andrew D. Turner, Florence R. I. Turner, Martha White, David Hartnell, Claire G. Crompton, Nicola Bates, Jan Egginton, Liz Branscombe, Adam M. Lewis, Benjamin H. Maskrey

**Affiliations:** 1 Centre for Environment, Fisheries and Aquaculture Science (Cefas), Barrack Road, Weymouth DT4 8UB, UK; 2 Budmouth Academy, Chickerell Road, Weymouth DT4 9SY, UK; 3 Veterinary Pathology Group, Unit 8, Temple Point, Bullerthorpe Lane, Leeds LS15 9JL, UK; 4 Veterinary Poisons Information Service, Godfree Court, 29-35 Long Lane, London SE1 4PL, UK

**Keywords:** cyanobacteria, cyanotoxins, anatoxin-a, dihydroanatoxin-a, dog poisoning, reservoir

## Abstract

Cyanobacterial blooms are often associated with the presence of harmful natural compounds which can cause adverse health effects in both humans and animals. One family of these compounds, known as anatoxins, have been linked to the rapid deaths of cattle and dogs through neurotoxicological action. Here, we report the findings resulting from the death of a dog at a freshwater reservoir in SW England. Poisoning was rapid following exposure to material at the side of the lake. Clinical signs included neurological distress, diaphragmatic paralysis and asphyxia prior to death after 45 min of exposure. Analysis by HILIC-MS/MS of urine and stomach content samples from the dog revealed the detection of anatoxin-a and dihydroanatoxin-a in both samples with higher concentrations of the latter quantified in both matrices. Detection and quantitative accuracy was further confirmed with use of accurate mass LC-HRMS. Additional anatoxin analogues were also detected by LC-HRMS, including 4-keto anatoxin-a, 4-keto-homo anatoxin-a, expoxy anatoxin-a and epoxy homo anatoxin-a. The conclusion of neurotoxicosis was confirmed with the use of two independent analytical methods showing positive detection and significantly high quantified concentrations of these neurotoxins in clinical samples. Together with the clinical signs observed, we have confirmed that anatoxins were responsible for the rapid death of the dog in this case.

## 1. Introduction 

Cyanobacteria are ubiquitous prokaryotic photoautotrophs, formally known as blue-green algae, attracting increased scientific and public attention, due in part to the suite of secondary metabolites they produce. Many of these low molecular weight compounds are toxic, causing adverse effects on humans, animals, plants and Protoctista [1,2,3]. Cyanotoxins can affect a variety of biological pathways, including hepato-, neuro-, cyto-, dermato-, geno-toxicity and carcinogenicity [4,5], with the neurotoxins including both anatoxins and saxitoxins. 

**Figure 1:**
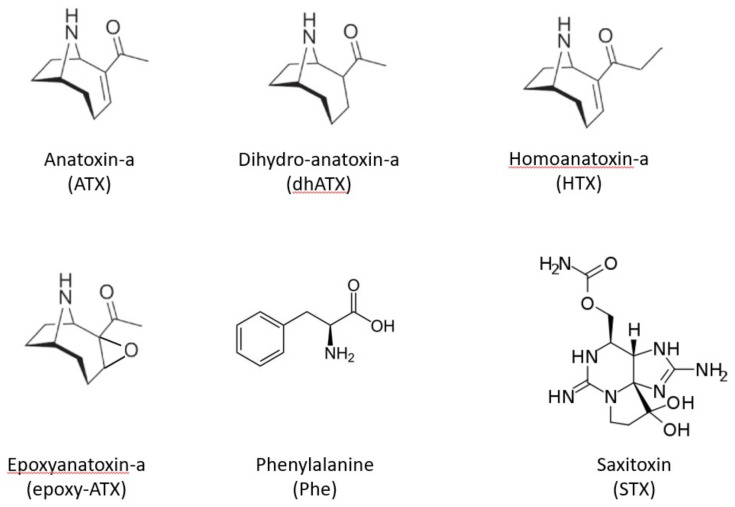
Chemical structures of example neurotoxic cyanotoxins including anatoxin-a and associated analogues, dihydroanatoxin-a, homoanatoxin-a and epoxyanatoxin-a, together with an interfering compound Phenylalanine and saxitoxin.

Saxitoxins (STX) are alkaloid neurotoxins, blocking voltage-gated sodium channels, causing nausea, leading to respiratory arrest and death in severe cases (Paralytic shellfish poisoning) [6]. Known STX producing cyanobacteria genera are *Aphanizomenon, Dolichospermum (Anabaena), Lyngbya, Cylindrospermopsis* and *Planktothrix.* Anatoxin-a (ATX), 2-acetyl-9-azabicyclo[4.2.1]non-2-ene [7], is a low molecular weight bicyclic secondary amine, neurotoxin that irreversibly binds to nicotinic acetylcholine receptors, causing loss of coordination, muscular fasciculations, convulsions and death by respiratory paralysis [1,8]. Known ATX producing cyanobacteria genera include *Dolichospermum (Anabaena), Aphanizomenon, Phormidium* and *Microcoleus* [9,10,11]. In addition to ATX itself, other analogues have been reported such as homoanatoxin-a (HTX), dihydroanatoxin-a (dhATX) and epoxyanatoxin-a (epoxy-ATX) [12,13,14,15,16,17]. (Figure 1).

In freshwaters, neurotoxins are prevalent and by extension, have attracted a high level of interest from the scientific community and water resource managers. Additionally, data suggests that human activities, namely global warming and eutrophication are expected to increase the number of cyanobacteria [18,19], therefore these toxins represent a serious threat to human, animal, and ecosystem health [3].

In addition to human health impacts through ingestion of drinking water and recreational exposure, neurotoxic blooms of cyanobacteria have caused animal poisonings globally for many years [1,3,18,20,21,22,23]. Animal deaths, due to the anatoxin-producing *Anabaena* were recorded in North America in the 1920s and 1950s [24]; the 1952 bloom at Lake Storm, Iowa, caused the deaths of several thousand birds and mammals. 

In Canada during the 1970s there were a series of cattle deaths attributed to ingestion of cyanobacterial scums. *Dolichospermum flos-aquae* (formerly *Anabaena flos-aquae*) was cultured and administered orally to calves to determine a minimum lethal dose of 360 to 480 mL of concentrated culture in a 60 kg animal. After 30 min under veterinary supervision, the calves suffered muscle fasciculations, paralysis and respiratory collapse and required intubation after 4 h, and they were maintained for 28 h before the experiment was terminated. The causative compound ATX was identified and isolated and found to have a LD_50_ of 50 µg kg^−1^ by MBA following intraperitoneal (i.p.) injection [25]. Other fatal animal poisonings linked to anatoxins have been reported in Canada, including cattle and waterfowl [26,27]. Dogs also show susceptibility to anatoxins poisoning. It is thought that the musty taste and odours of cyanobacteria attract the attention of dogs scavenging habits, and they like to swim allowing cyanobacteria to stick to fur followed by ingestion or through grooming [28]. An investigation into dog and other animal deaths near a lake in South Dakota, reported a bloom dominated by *Dolichospermum flos-aquae*, and identified ATX-a as the causative compound by HPLC. [25]. Benthic cyanobacteria were also accountable for two dog deaths from a river in France, with ATX identified from *Phormidium favosum*, by liquid chromatography coupled to tandem mass spectrometry [22]. Anatoxins in Finnish lakes have also been linked to reports of animal poisonings including both cattle and dogs [29,30], as well as reports of ATX and dihydroanatoxin-a (dhATX) being linked to dog fatalities in Germany [15]. Outside Europe, in the US, between 2007 and 2011, as part of the Harmful Algal Bloom-related Illness Surveillance System (HABISS), 67 cases of canine cyanotoxicosis events in freshwaters were reported, 58% were fatal and of those 32% were ascribed to ATX [21]. Multiple reports also exist of anatoxin-producing cyanobacteria in New Zealand water bodies, resulting in dog deaths [17,31,32].

Closer to the UK, ATX has been reported in several freshwater lakes in Ireland, with links to fatal canine poisonings in multiple regions [33,34,35]. In the UK itself, five cases of cyanotoxicosis in dogs were reported after drinking from a Loch in Scotland; four cases were fatal by neurotoxicosis within 30 min. The causative cyanobacterium was discovered to be benthic *Oscillatoria*. ATX was identified and extracted from shoreline samples, stomach contents and cultured *Oscillatoria*, using liquid and gas chromatography [36,37]. 

Routine monitoring is not conducted in inland UK water bodies, so hazard assessment involves primarily the responsive microscopy testing of water samples following reports of cyanobacterial presence from public or water-body owners, with action taken to restrict water access when cell densities exceed threshold limits [38]. Other schemes exist including the citizen science reporting of potentially toxic cyanobacterial blooms throughout the country, which can help publicise the presence of potentially toxic blooms [39]. Nevertheless, without a systematic risk management system in place, the cyanobacterial blooms occurring on a regular basis during the warmer months of the year, are likely to continue to result in exposure incidents affecting animal as well as potentially human health. Indeed, multiple reports of dog poisonings following cyanobacterial-exposure are found every year in the UK media and news reports, e.g., [40,41,42,43], which has resulted in a range of advisory services published on-line to warn owners of the risks from blue-green algae in some freshwater locations [44,45,46].

In June 2022, an incident was reported involving the suspected poisoning of a dog at a freshwater reservoir in SW England. The dog had been one of seven dogs in a visiting group, walking along the side of the water body. The affected dog was observed to lick or nibble upon some stranded material including a dead fish at the shoreline. Symptoms of paralysis were observed within minutes of exposure, with death of the dog occurring after 45 min. Consequently, an investigation was conducted to establish the cause of death with a view to determining if cyanobacterial neurotoxins presented a risk to animal and human health. Specifically, analysis was conducted to establish the potential presence of either anatoxins and/or saxitoxins in postmortem samples obtained from the deceased animal.

## 2. Results

### 2.1. Incident

**Figure A1:**
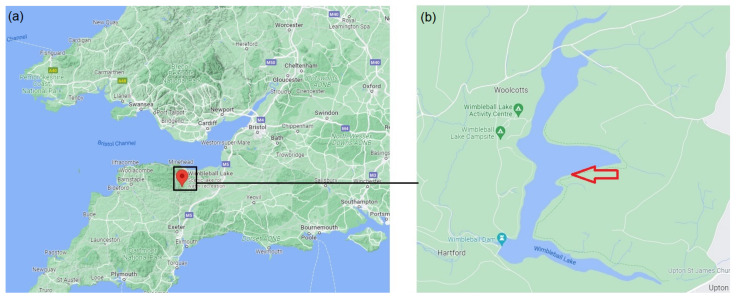
Maps showing (**a**) area in relation to SW England and Wales and (**b**) close up of reservoir with red arrow showing location where dog poisoning occurred (map images courtesy of Google Earth).

A male dog aged 2 years and 4 months and weighing 32 kg was one of seven dogs (all Flatcoat Retrievers) that were walked at a freshwater reservoir (Wimbleball Lake) in Somerset, SW England. The location was on the eastern lake shore at the southern end of an east-facing cove (map shown in Appendix A Figure A1). Coordinates of the incident location were 51°03′45.0″ N, 3°27′54.0″ W. On the 8 June 2022, all the dogs had been walking along the side of the lake. The affected dog was seen at one point to lick or nibble on material that was present on the ground, close to the water’s edge. On realising this was close to one of three dead fish, the dog was quickly removed from the location of the fish. Shortly afterwards, the dog showed signs of neurological distress, including apparent limb paralysis. The dog was unable to move and had signs of dyspnoea, consequently exhibiting diaphragmatic paralysis and asphyxia. No gastro-intestinal signs were observed such as diarrhoea and vomiting. After approximately 30 min, with signs of extensive paralysis, breathing difficulties and loss of pulse, cardiopulmonary resuscitation (CPR) was administered by two people but when no signs of life were obtained after a further 15 min, resuscitation was halted and the dog presumed dead. The animal was then taken immediately to a nearby veterinary practice where it was confirmed as dead on arrival. None of the remaining six dogs exhibited any signs of illness at the time or since and remain healthy. One of the two people performing CPR, including mouth to mouth ressusitation, at the scene later (>12 h) became unwell, with diarrhoea and stomach cramps, but with no apparent neurological symptoms. Symptoms resolved within 24 h. 

### 2.2. Post-Mortem Investigation

Autopsy revealed no gross visual signs of abnormalities present in any organs. No typical signs of cyanobacterial toxicosis were observed, including the absence of diarrhoea and vomiting, but analysis did not preclude cyanotoxin-triggered peracute fatality. The stomach contents were analysed for a wide range of volatile organic compounds by GC-MS. Analysis revealed no positive detection of any drugs, poisons, toxins or chemical pollutants that were incorporated into the detection method. General classes of compounds incorporated into the method database comprising over >50,000 compounds included selected drugs of abuse, pharmaceuticals, veterinary drugs, industrial chemicals, pesticides, some organic compounds and plant toxins. There was also no detection of other miscellaneous compounds such as caffeine or theobromine. Whilst there was no detection of any of the 50,000 compounds, the method did not incorporate all drugs of abuse, ethylene glycol, neuromycotoxins, aflatoxins, alphachloralose or warfarin and coumarin derivatives. Samples (stomach contents, urine, blood and a blood clot) were submitted to Cefas for further analyses.

### 2.3. Toxin Analysis

#### 2.3.1. LC-MS/MS

Reverse-phase liquid chromatography with tandem mass spectrometry (LC-MS/MS) conducted using method 1 (see Section 4 Methods and materials) for the targeted determination of lipophilic cyanotoxins including microcystins and nodularin showed no detected toxins following the analysis of all four clinical samples. Similarly, MS/MS acquisition showed no peaks evidencing the presence of either STX or CYN. In addition, a full HILIC-MS/MS method for saxitoxins was also applied, but no other STX analogues were detected (data not shown). For ATX, however, chromatographic peaks were observed in the urine and stomach content extracts, although these were found to elute very early on the chromatogram (data not shown), so could not be relied upon for accurate quantitation. Consequently, the samples were re-analysed using method 2, the refined method using Hydrophilic Interaction Liquid Chromatography with tandem mass spectrometry (HILIC-MS/MS) which resulted in improved chromatographic retention on-column for the hydrophilic cyanotoxins. 

**Figure 2:**
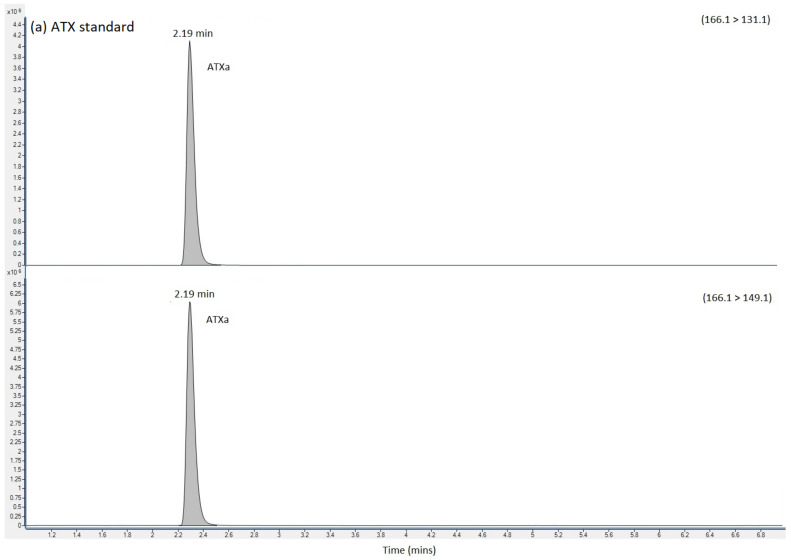

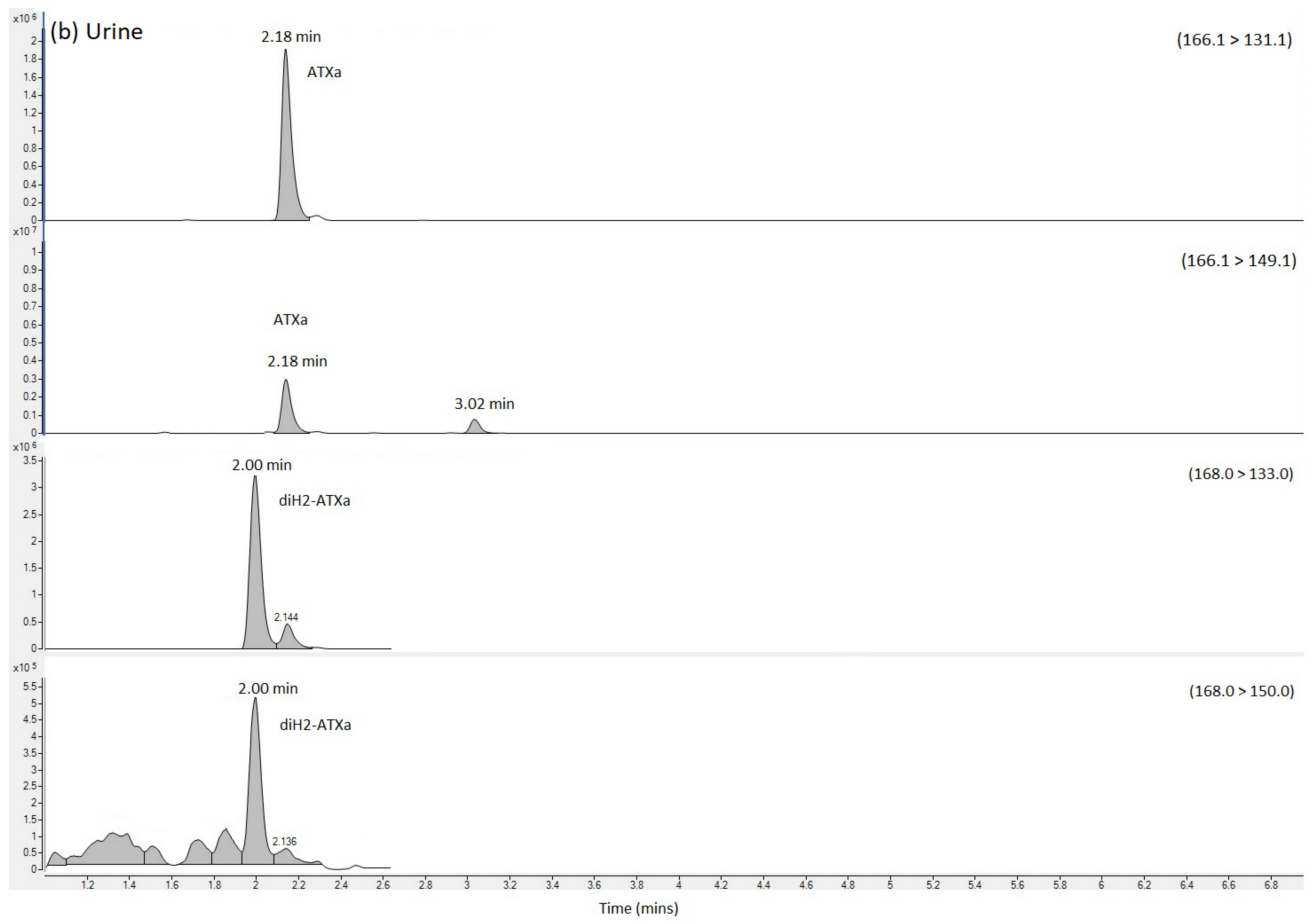

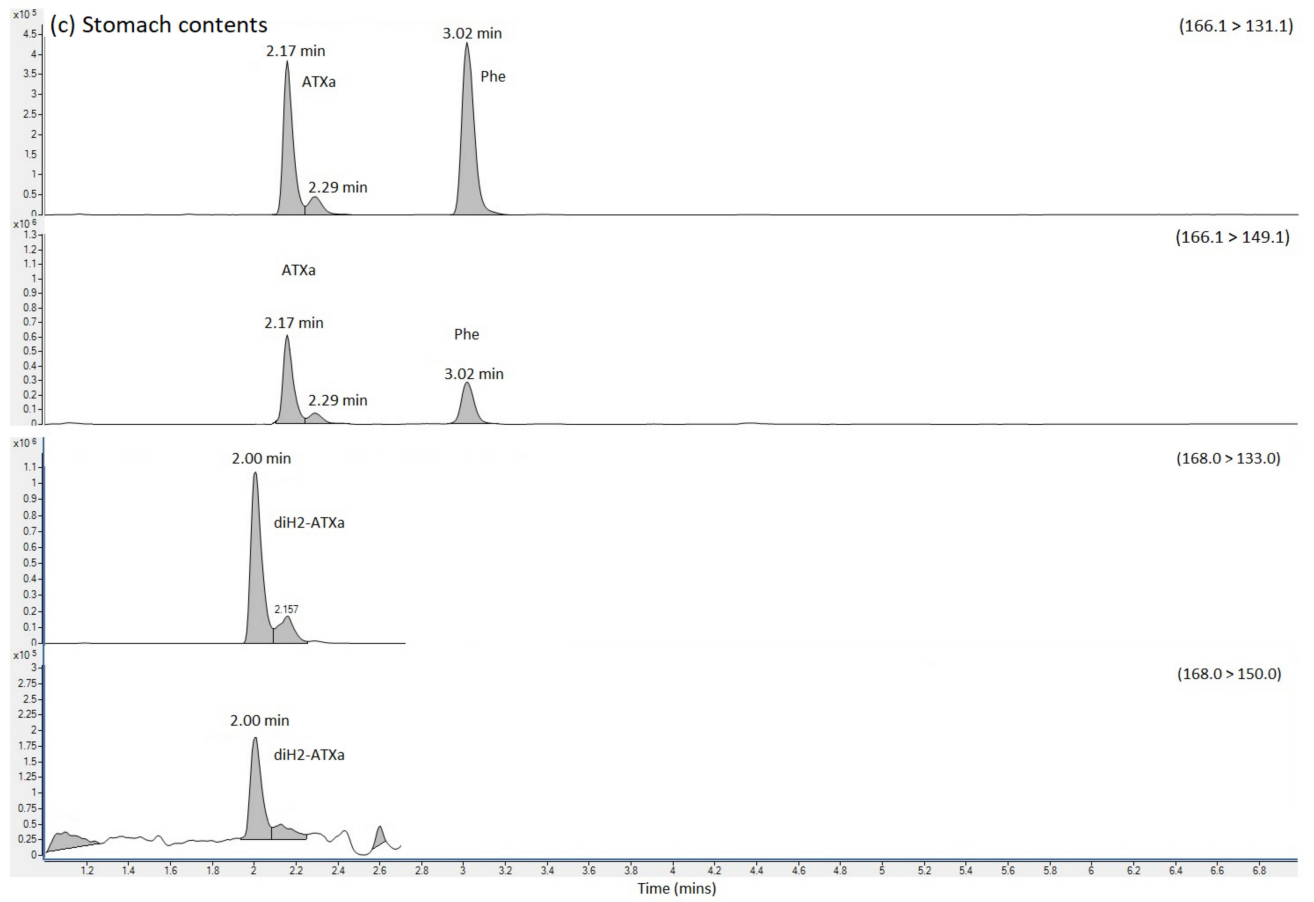

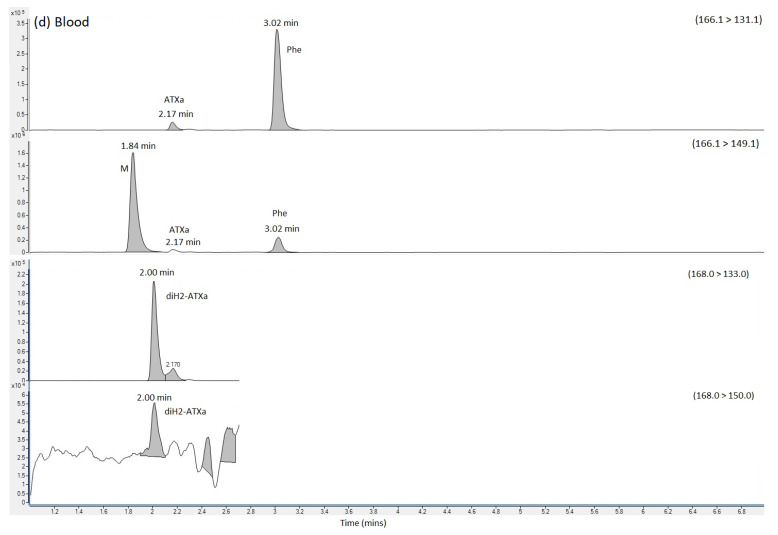
SRM chromatograms obtained following the analysis of (**a**) ATX calibration standard (**b**) urine extract (**c**) Stomach content extract and (**d**) blood extract, labelling SRM transitions and chromatographic retention times for ATX, dhATX and Phe. ATX = anatoxin-a; dhATX = dihydro anatoxin-a; Phe = phenylalanine; M = matrix interference peak.

HILIC-MS/MS analysis following method 2 showed no detected peaks for CYN or STX, so there was no evidence for either of these cyanotoxins in any of the four clinical samples. Selected reaction monitoring (SRM) peaks for ATX were, however, again detected primarily in the urine and stomach extracts. Small SRM peaks were also detected for ATX in the blood samples, but at levels below the limit of quantification. Figure 2 displays the SRM chromatograms for ATX present in the pure ATX reference standard, alongside SRM chromatograms obtained following analysis of the urine, stomach content and blood extracts. A single SRM peak was observed at 2.19 min in the standard, with peaks detected at 2.17 to 2.18 min in the clinical samples, with retention times within 1% of each other. HILIC-MS/MS analysis of the biogenic amino acid phenylalanine (Phe), isobaric to ATX, showed elution of the chromatographic peak at 3.02 min, with total resolution from ATX. Consequently, the ATXs detected using the method was free from interferences from Phe, with the latter also detected in stomach content and blood extracts. Ion ratios for the ATX SRM peaks in the clinical samples were found to be within 10% of the ratios determined in the ATX standard.

SRMs were also obtained for other anatoxin analogues, with positive detection of dhATX in the urine, stomach contents and to a lesser extent, the blood extract samples (Figure 2).

Quantitation of ATX was conducted against external calibration standards prepared from dilutions of certified reference standards. Table 1 summarises the concentrations determined showing concentrations of 599 ng ATX per mL of urine and 1044 ng/g ATX in stomach contents using the HILIC-MS/MS method. The ATX analogue dhATX was quantified at much higher concentrations when quantifying against the ATX calibration; 5494 ng/g in urine and 21,008 ng/g stomach contents. Small dhATX SRM peaks were also detected in both the blood sample and the blood clot but at levels below the limit of quantitation. In the absence of a dhATX standard these calculations are based on the assumption that both compounds perform identically in the mass spectrometer.

#### 2.3.2. LC-HRMS

**Figure 3:**
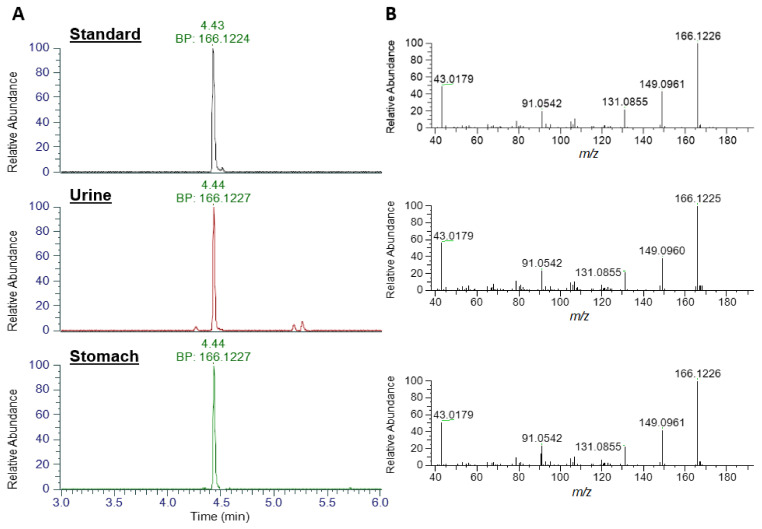
LC-HRMS identification of ATX. (**A**) Base peak (BP) Extracted Ion Chromatograms at *m*/*z* 166.1226 and (**B**) product ion scans for anatoxin standard, urine extract and stomach extract.

An extracted ion chromatogram (XIC) of ATX standard at *m*/*z* 166.1226 (± 5 ppm) exhibited a strong peak at 4.43 min with a base peak of *m*/*z* 166.1224, consistent with the singly protonated form of ATX (Figure 3). Analysis of urine and stomach extracts revealed identical peaks at 4.44 min, with base peaks (BP) of *m*/*z* 166.1227, evidencing the presence of ATX in these extracts. Product ion scans of standard and extracts revealed identical fragmentation patterns, with dominant daughter ions at *m*/*z* 149.0961, 131.0855, 91.0542 and 43.0179, consistent with those daughter ions used in the HILIC-MS/MS method and described elsewhere [15].

**Figure 4:**
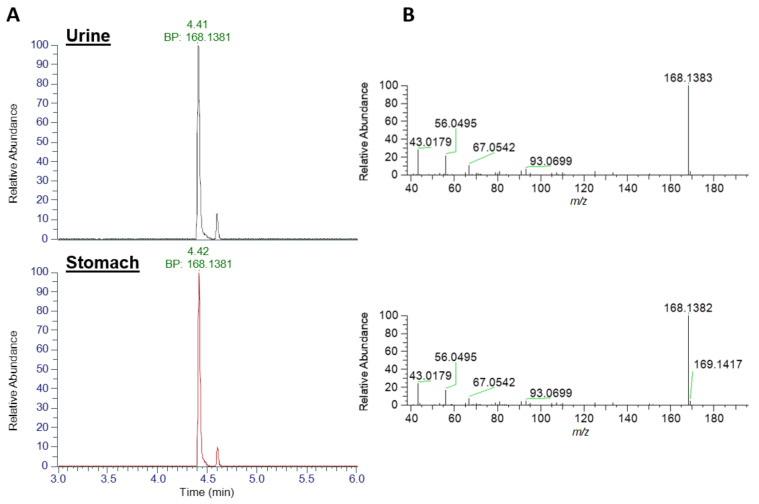
LC-HRMS identification of dhATX. (**A**) Base peak (BP) Extracted Ion Chromatograms at *m*/*z* 168.1383 and (**B**) product ion scans for urine extract (top traces) and stomach extract (bottom traces).

Analysis for dhATX (Figure 4), revealed an earlier eluting chromatographic peak at 4.41 min with an XIC of *m*/*z* 168.1383 in both urine and stomach extract, with base peaks identified at *m*/*z* 168.1381. No standard was available for comparative analysis. Fragmentation patterns showed matching daughter ions of *m*/*z* 43.0179, a shared daughter ion with ATX and also *m*/*z* 56.0495. Both of these fragmentation patterns are consistent with those described in [47]. As detailed in Table 2, mass error between the measured and theoretical masses of the parent ion were <2 ppm (0.602 for ATX and −1.189 for dhATX) further confirming their identification. 

Quantitation of ATX and dhATX determined values of 977 and 8,165 ng/mL urine, and 891 and 20,637 ng/g of the stomach contents. These concentrations were comparable with those obtained by the HILIC-MS/MS method (Table 1), particularly with the elevated levels of dhATX. The use of two independent analytical measurements provides confidence of the values obtained in these samples. Low levels of dhATX below the limit of quantitation (<LOQ) of the calibration range were detected in both the clot and blood samples.

#### 2.3.3. LC-HRMS Minor Analogues

**Figure 5:**
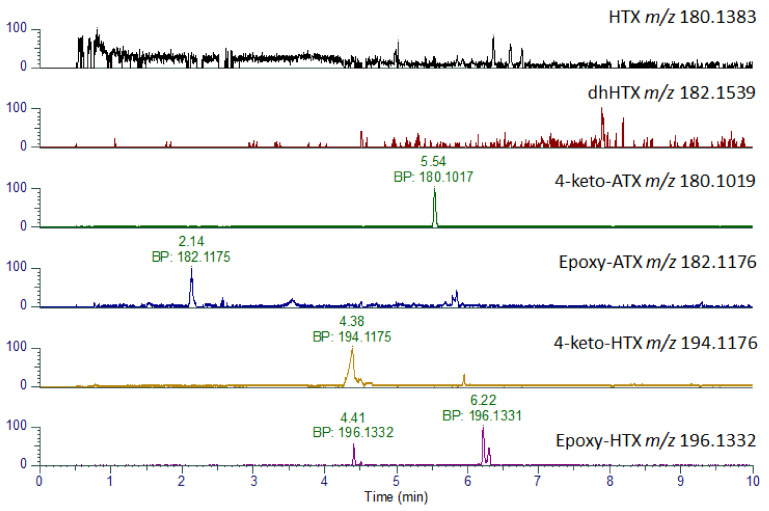
LC-HRMS identification of ATX analogues. Extracted Ion Chromatograms for anatoxin analogues obtained in stomach extract showing base peaks (BP) and associated accurate masses.

In addition to ATX and dhATX, a number of other analogues were identified including 4-keto-ATX, 4-OH-ATX and epoxy-ATX, as well as their methylated homo analogues [17,47,48,49]. As shown in Table 3, some of these analogues such as HTX and 4-keto-ATX share the same nominal mass and so are difficult to differentiate on LC-MS/MS systems in the absence of authentic standards. However, due to their differing chemical formulae it was possible to distinguish these by LC-HRMS. As the stomach content sample had the highest levels of ATX and dhATX, this sample was used to investigate the presence of these six additional analogues (Table 3). As shown in Figure 5 and Table 3, peaks consistent with the expected parent masses were identified for 4-keto-ATX (5.52 min), 4-keto-HTX (4.47 min), epoxy ATX (2.14 min) and several peaks for epoxy-HTX (4.46, 6.21 min). All these peaks have a mass error of −1.11 ppm or less indicating good confidence in their identification. No peaks were identified for HTX, or diH2-homo ATX (dhHTX). In the absence of analytical standards for these compounds, fragmentation spectrum for these compounds were obtained (Figure S1) in an attempt to confirm their identity. Both 4-keto-ATX (Figure S1A) and 4-keto-HTX (Figure S1C) generated daughter ions consistent with a loss of 59 amu at *m*/*z* 121.0647 and 135.0440, respectively. Fragmentation of epoxy ATX (Figure S1B) and epoxy-HTX (Figure S1D) generated ions consistent with the loss of H_2_O at *m*/*z* 164.1072 and 178.1223, respectively as expected from the loss of the epoxide group. Both compounds also produced daughter ions at *m*/*z* 56.0495, with epoxy ATX also producing a fragment at *m*/*z* 43.0179. These daughter ions are comparable to that reported by [47], although due to the lack of other shared daughter ions, definitive identification of these compounds cannot be assigned.

## 3. Discussion

Incidents involving the poisonings of dogs following exposure to anatoxins whilst not routinely confirmed, have been reported worldwide in recent years. Whilst many reports exist in the media, few of these have been scientifically linked to cyanobacterial sources and verified through examination of clinical or post-mortem samples. Nevertheless, dog deaths have on occasions been unambiguously linked to the presence of anatoxins, including ATX and associated analogues (dhATX, HTX) in water and cyanobacterial sources such as benthic mats [15,50]. Anatoxins are also known to be distributed globally, throughout a wide range of climatic environments. In Europe, 25% of German waterbodies were found to contain ATX, with toxin presence also confirmed in Finland, Ireland and France, with the highest toxin prevalence occurring in benthic cyanobacterial mats, as opposed to within surface waters [16,22,33,34,51,52].

In this study, a 32 kg male dog exhibited signs of paralysis within 10 min of suspected oral exposure to a dead fish and/or other nearby biota situated on the shore of a freshwater reservoir in SW England in June 2022. Death occurred within 45 min of exposure, despite attempts at CPR. The dog was one of seven present in the location, all of whom had played along the shoreline, with the affected dog being the only one observed to have encountered the dead fish. The other dogs exhibited no signs of sickness or distress. Anatoxins are known to be rapidly absorbed and distributed throughout the body after exposure. Administration of lethal doses of ATX in animal studies results in death within minutes due to muscular paralysis and asphyxiation [53,54], which fits well with the observations in this case [16]. Consequently, the symptomology and poisoning showed evidence for ingestion of anatoxins, with the source of the toxins likely to be the fish and/or other surrounding biota, as opposed to direct exposure to toxins in the water.

Correlating concentrations of toxin in post-mortem samples to toxicity exposure levels is very difficult, given the lack of data regarding metabolism, including absorption and excretion rates as well as potential biotransformation reactions. In terms of lethal doses of anatoxins, the LD_50_ for oral ingestion has been suggested as >5 mg/kg body weight [16], and between 1–10 mg/kg body weight [53,54] which for a 32kg dog equates to >32–320 mg of ATX. The toxin concentrations quantified in this study using HILIC-MS/MS were approximately 0.6 mg/mL and 1.0 mg/g of ATX and 6.0 mg/mL and 21 mg/g in urine and stomach contents, respectively (Table 3). Noting the sample amounts received were 2.16 mL urine and 0.083 g stomach contents, representing a small proportion of the total samples originally taken, these concentrations equate to approximately 1.3 mg (urine) and 0.08 mg (stomach) of ATX, with 13 mg (urine) and 1.75 mg (stomach) of dhATX. With dhATX shown to be more than four times more toxic than ATX [17], the combination of both toxins equates to a total of approximately 60 mg of ATX equivalents. Given that other ATX analogues were also detected, albeit with unknown toxicity equivalents, and the toxins would have likely distributed to other tissues following absorption, and that the sample sizes received at Cefas were only sub-samples of larger sample volumes used for general extensive toxicological screening including organic contaminants, there is strong evidence that the toxins were ingested at levels within the 32–320 mg range of LD_50_ limits for ATX. Such doses fit with multiple literature reports where high concentrations of ATX have been quantified in many different species of cyanobacteria, with ATX concentrations reaching 13 mg/g cyanobacteria in laboratory cultures and environmental samples from Finland and Canada [10,55], as well as other examples quantified in environmental samples from France, Ireland, New Zealand, Germany, Kenya and Iran [16]. A summary of ATX concentrations quantified to date in dog stomach samples is summarised in Table 4, alongside detection of other ATX analogues, and concentrations in related environmental samples. As shown, concentrations were reported in approximately half of the samples analysed, with values varying hugely. ATX concentrations reach as high as 36 mg/g stomach contents in samples from New Zealand, over 30,000 times higher than levels quantified in this study. Conversely, the stomach contents of dead dogs from Germany contained as low as 0.025 µg/g ATX, significantly lower than the amounts determined here.

Clinical samples from the deceased animal included urine, blood, a blood clot of unknown origin and stomach contents. These were extracted and analysed using two different mass spectrometric methods; HILIC-MS/MS for targeted quantitation and LC-HRMS for accurate mass confirmation and quantitation. Reverse-phase LC-MS/MS, used for analysis of lipophilic cyanotoxins, was also modified for detection of hydrophilic toxins, but retention times of these target analytes were too low, so quantitation was not performed. SRMs generated by HILIC-MS/MS for hydrophilic cyanotoxins showed no detectable peaks for CYN or any saxitoxins, but did show the clear presence of ATX, most notably in the urine and stomach content extracts. Chromatographic separation between ATX and Phe was achieved with the refined HILIC gradient, removing the potential for false detection or over-estimation of the former, given the shared SRM transitions associated with both compounds due to the isobaric nature of both precursor and product ions [62] and previous false reports of human fatality published following LC-MS detection of Phe [63]. SRMs for other known ATX analogues were also generated, with none detected with the exception of clear SRM peaks for dhATX in the urine and stomach samples. Further confirmation of ATX and dhATX was obtained through the LC-HRMS analysis of the same extracts, with evidence generated for XIC detection of both ATX and dhATX in all four samples, with the urine and stomach extracts showing concentrations within the calibration range of the method. Specific confirmation was generated through both accurate mass detection of expected base peak ions and with the confirmation of product ions matching those utilised for targeted HILIC-MS/MS detection and those reported previously in the literature. The LC-HRMS approach also allowed differentiation between the isobaric ATXs and Phe, through acquisition of readily distinguishable [M+H]^+^ XICs at 166.1154 and 166.0790, respectively [17,63]. Notably, the results demonstrated the high prevalence of dhATX in the clinical samples, with the urine and stomach contents containing ten times the concentrations in comparison to ATX (Table 3). These findings agree with those reported recently in Germany following canine fatalities linked to consumption of benthic cyanobacterial mats [15] as well as prior reports suggesting dhATX is the congener most likely to cause dog deaths [31]. DhATX is known to present intracellularly within cyanobacteria, evidencing natural production [17,64], but is also considered as the major degradation product of ATX. The tentative identification of the epoxy and keto ATX degradation products further confirms the presence of ATX in these samples, and highlights the requirement for additional analytical standards for these compounds to aid in confirmation and quantitation.

To date, the maximum concentration of ATX dissolved in lake waters has been 444 µg/L, in samples taken from a lake in Ireland [33]. Whilst it is not inconceivable for concentrations to exceed this level, given the absence of routine monitoring for these toxins with only occasional research surveys conducted, it seems highly unlikely that toxin levels were high enough in the water to result in poisoning through drinking lake water alone. As shown in previous studies where extremely high concentrations of toxins were confirmed in benthic cyanobacterial mats [15,32,65,66], it seems that in this study the exposure occurred through contact with such material, including potentially cyanobacterial debris present on the surface of the dead fish. The absence of any illness in the other dogs at the lake that had only contact with the reservoir water, certainly seems to support the hypothesis that the affected animal was exposed to much higher doses of toxins, noting that cyanobacterial mats and scums can contain a thousand to a million-fold increase in cyanobacterial cell numbers in comparison to normal cyanobacterial cell populations [67]. Indeed, it was noted at the time of the incident that contact with some kind of material at the edge of the water was seen, so potentially this may have been ingested. Unfortunately, water samples were not taken at the time of the incident by the authorities, and on returning to the scene to collect the fish, the remains had been removed. Given the incident was not notified to Cefas until a month later, water samples were not taken given that the samples would not have been representative of the water present at the time of the poisoning, particularly as rapid decay of ATXs has been reported in biological and environmental matrices [63,67,68]. Similarly, no collection of algae was made after the incident to help support the hypothesis that benthic cyanobacteria may have been a cause. However, from the evidence collated here, there is clear confirmation of anatoxin poisoning in the dog as a consequence of exposure to the neurotoxins at the lakeside.

Consequently, the chemical analysis conducted using two independent mass spectrometric methods provided excellent evidence confirming the hypothesis of ATX and dhATX-related poisoning in the affected dog, with the likely source being material present on or within the dead fish at the water’s edge, as opposed to toxins dissolved in the lake water. 

## 4. Methods and Materials

### 4.1. Animal Autopsy, Toxicology and Samples

The full clinical history of the dog was assessed at a local veterinary practice. This incorporated the assessment of the incident and potential exposure scenarios, followed by post-mortem assessment. Clinical samples obtained during the post-mortem examination included stomach contents, urine, blood and a blood clot of unknown origin. Clinical samples were utilised for toxicity testing. Stomach contents from the deceased dog were assayed using Gas Chromatography with Mass Spectrometry (7250 GC-MS) (Agilent, Manchester, UK) following extraction by a modified QuEChERS, (Quick, Easy, Cheap, Effective, Rugged, and Safe) extraction procedure as published by [69]. The procedure facilitated the identification of a wide range of drugs and poisons including, but not limited to, barbiturates, anticonvulsants, analgesics, antihistamines, hypnotics, some drugs of abuse and recreational drugs, tranquillizers, muscle relaxants, and toxins including metaldehyde and strychnine. In addition to human and veterinary drugs, industrial products, pesticides (including organophosphates, pyrethroid, carbamates, and fungicides), plant derived toxins and other natural products were included in the screen. 

### 4.2. Chemicals and Reagents for Toxin Analysis

Instrument solvents used for preparation of mobile phases were of LC-MS-grade (Fisher Optima, ThermoFisher, London, UK) and all chemicals were LC-MS reagent grade where possible, otherwise HPLC grade. Sample preparation reagents were all HPLC grade. Cyanotoxin reference toxin standards comprising the microcystin analogues (MC-RR, MC-LA, MC-LY, MC-LF, MC-LW, MC-YR, MC-WR, MC-HilR, MC-HtyR, MC-LR, [Asp3] MC-LR), Nodularin (Nod), ATX, Cylindrospermopsin (CYN) were all obtained from Enzo Life Sciences (Enzo, Exeter, UK), with secondary calibration check standards and Saxitoxin (STX) standard prepared using certified reference solutions from Biotoxin Metrology, National Research Council of Canada (NRCC, Halifax, Nova Scotia, Canada). Enzo reference standards received as solid powders were dissolved in suitable volumes of 50% aqueous methanol, to form stock solutions. A mixed stock solution was prepared by combining aliquots of each stock, followed by a seven-level suite of working calibration solvent standards resulting in a calibration range between 1.0 ng/mL to 500 ng/m per toxin. Phe was also obtained from Merck (Poole, UK) and used for confirmation of resolution from ATX, given the isobaric nature of the two compounds and the potential for false positive detection of ATX in matrices containing Phe [62].

### 4.3. Toxin Analysis

#### 4.3.1. Sample Processing

Post-mortem samples provided from the dog for toxin testing consisted of 2.16 mL of urine, trace levels of a blood clot (0.041 g) and stomach contents (0.083 g), and 1.56 g of blood. These had been stored under refrigerated conditions until shipment and receipt at Cefas. Given the small amounts of samples available for testing, one single extraction was performed on each sample, using 50% aqueous methanol (MeOH), to target extraction of both hydrophilic and lipophilic cyanotoxins [70]. The urine and blood samples were both diluted 1:1 (*v/v*) with 50% MeOH, with the trace amount of clot and stomach content tissues weighed and 2.0 mL 50% MeOH added. The contents of all four samples and solvent were shaken (vortex mix, 2500 rpm, 90 s), before centrifugation (4500 rpm; 10 min; 20 °C). Post-centrifuged supernatants were filtered through 0.45 µm Teflon syringe filters (Merck, Poole, UK) into glass LC-MS autosampler vials and submitted for toxin analysis.

#### 4.3.2. LC-MS/MS

Targeted analysis of sample extracts was conducted using ultra-high performance LC-MS/MS. Two different methods were employed for detection of all cyanotoxins of interest.

Method 1 (LC-MS/MS) involved the use of reverse-phase UHPLC for analysis of lipophilic cyanotoxins, specifically the microcystins and nodularin, following the validated and accredited method of [70]. An Agilent (Manchester, UK) 6495B triple quadrupole mass spectrometer (MS/MS) coupled to an Agilent 1290 Infinity II UHPLC system was used for LC-MS/MS analysis. Chromatography was conducted using a 1.7 μm, 2.1 × 50 mm Waters (Manchester, UK) Acquity UPLC BEH C18 column (P/N 186002350) in conjunction with a Waters (Manchester, UK) VanGuard BEH C18 1.7 μm, 2.1 × 5 mm guard cartridge (P/N 186003975). The columns were held at +60 °C with samples held in the autosampler tray at +10 °C. The sample injection volume was 5 μL and the mobile phase flow rate was 0.6 mL/min. Mobile phase A consisted of water + 0.025% formic acid, mobile phase B comprised acetonitrile (MeCN) + 0.025% formic acid. The UHPLC gradient was a modified version of [70]: 5% B initial conditions holding for 0.75 min, rising to 25% B at 1.0 min holding until 2.0 min, rising to 40% B at 3.5 min, increasing further to 50% B at 4.5 min, a quick rise to 95% B at 4.6 min and held until 6.0 min until dropping back to 5% B at 6.1 min. The total run time was 6.5 min. Each instrumental sequence started with a series of instrumental blanks, followed by toxin calibration standards. After running sample extracts, instrumental sequences finished with a second set of calibration standards. In addition to the validated quantitative determination of MCs and Nod, injections were performed for ATX and CYN noting that LC peaks were observed to elute in the dead volume and that a different method would be required for detecting these hydrophilic cyanotoxins more appropriately. The MS/MS tune parameters were as follows: 150 °C source temperature, 400 °C sheath gas temperature, 15 L/min desolvation gas flow, 50 psi nebuliser gas, 12 mL/min sheath gas flow, Capillary voltage was held at +1.0 kV, with 0 V nozzle voltage and I-Funnel voltages set to 150 V and 80 V for the high pressure and low pressure RF, respectively. Selected reaction monitoring (SRM) transitions were built into the MS/MS method using positive mode acquisition for each toxin. Parent and daughter ions, as well as cone and collision voltages were based on those described by [70]. The LC-MS/MS MC and Nod method involved the direct quantitation of toxins against working standards. Quantitation was performed using external calibration and results calculated in terms of ng/mL of sample extracts and extrapolated to toxin concentration within tissue depending on the extraction dilution factor applied.

Method 2 (HILIC-MS/MS) utilised the same Agilent LC-MS/MS system and MS source conditions, but higher capillary voltage of +2.5 kV and with chromatographic separation performed using hydrophilic interaction liquid chromatography (HILIC). This was applied to the targeted detection of the hydrophilic ATX, CYN and STX in sample extracts, with any positive detection quantified against calibration working standards. The HILIC LC method was based upon the method developed and interlaboratory-validated for determination of saxitoxins in shellfish [71]. HILIC involved the use of an Agilent Poroshell HILIC-Z analytical column (2.7 µm, 120 HILIC-Z 2.1 × 100 mm UHPLC). The column was held at +30 °C with samples held in the autosampler tray at +10 °C. The sample injection volume was 2 μL and the mobile phase flow rate was 0.5 mL/min. Mobile phase A consisted of 500 mL water + 75 µL formic acid and 300 µL ammonium hydroxide, with mobile phase B comprising 900 mL acetonitrile (MeCN) + 100 mL water + 100 µL formic acid. The UHPLC gradient was a refined version of [71] Turner et al., 2020, optimised to improve chromatographic retention of ATX and CYN. The gradient started with 100% B initial conditions holding for the first 0.4 min, decreasing slightly to 97% B1 at 1.0 min, dropping further to 75% B at 4 min, continuing to 50% B at 4.5 min, holding until 5.5 min, then reverting back to 100% B at 6.0 min. The total run time was 7.0 min.

Selected Reaction Monitoring (SRM) transitions, together with associated collision energies are summarised in Table 5 as applied to each method. SRM transitions were selected for compounds available as reference standards and were previously optimised through MS source infusion experiments [70]. SRM transitions for additional anatoxin analogues were selected from literature reports of published method [52]. During set-up and validation of the methods, precision of the instrumental method was assessed, through the repeat injection of both standards and contaminated algal extracts. Precision was found to be <3% within a sequence, showing excellent injection repeatability. Additional analysis was conducted for the full suite of STX analogues, with SRM transitions and associated collision energies detailed in [69].

#### 4.3.3. LC-HRMS

LC-High Resolution Mass Spectrometry (LC-HRMS) was conducted using an Orbitrap Exploris 120 mass spectrometer coupled to a Vanquish UPLC system (Thermo Fisher, Hemel Hempstead, UK). Toxins were separated on a Acquity UPLC HSS T3 1.8 µm 2.1 × 100 mm column (Waters, Manchester, UK) maintained at 30 °C. Mobile phase A was 100% H_2_O, and B was 100% acetonitrile, both containing 0.1% formic acid. LC flow was maintained for 2 min at 100% A, followed by an increase to 90% B at 10 min, held for 1 min before returning to starting conditions at 11.5 min, with an overall cycle time of 15 min. Flow rate was 400 µL/min and the injection volume was 1 µL. LC effluent from 0.5–10 min was directed into the H-ESI source of the mass spectrometer which was operating in positive ion polarity with a voltage of 3500 V, sheath gas 50, sweep gas 1, ion transfer tube temp of 325 °C and vaporiser temp of 350 °C. The orbitrap was set to perform a full scan at a resolution of 60,000 with a scan range of *m*/*z* 100–200 using an internal lock mass calibrant of *m*/*z* 203.0855. To obtain fragmentation spectra, product ion scans were performed for parent ions with collision energies of 10, 20 and 40 V and orbitrap resolution of 15,0000. Quantitation of ATX and dhATX was performed from the full scan data using extracted ion chromatograms of masses as detailed in Table 2, utilising the same standard curve as used for HILIC-MS/MS. Data was acquired using Xcalibur v4.5 (Thermofisher, Hemel Hempstead, UK) and processed using FreeStyle 1.8 (Thermofisher, Hemel Hempstead, UK) and TraceFinder 5.1 (Thermofisher, Hemel Hempstead, UK). 

LC-High Resolution Mass Spectrometry (LC-HRMS) was conducted using an Orbitrap Exploris 120 mass spectrometer coupled to a Vanquish UPLC system (Thermo Fisher, Hemel Hempstead, UK). Toxins were separated on a Acquity UPLC HSS T3 1.8 µm 2.1 × 100 mm column (Waters, Manchester, UK) maintained at 30 °C. Mobile phase A was 100% H_2_O, and B was 100% acetonitrile, both containing 0.1% formic acid. LC flow was maintained for 2 min at 100% A, followed by an increase to 90% B at 10 min, held for 1 min before returning to starting conditions at 11.5 min, with an overall cycle time of 15 min. Flow rate was 400 µL/min and the injection volume was 1 µL. LC effluent from 0.5–10 min was directed into the H-ESI source of the mass spectrometer which was operating in positive ion polarity with a voltage of 3500 V, sheath gas 50, sweep gas 1, ion transfer tube temp of 325 °C and vaporiser temp of 350 °C. The orbitrap was set to perform a full scan at a resolution of 60,000 with a scan range of *m*/*z* 100–200 using an internal lock mass calibrant of *m*/*z* 203.0855. To obtain fragmentation spectra, product ion scans were performed for parent ions with collision energies of 10, 20 and 40 V and orbitrap resolution of 15,0000. Quantitation of ATX and dhATX was performed from the full scan data using extracted ion chromatograms of masses as detailed in Table 2, utilising the same standard curve as used for HILIC-MS/MS. Data was acquired using Xcalibur v4.5 (Thermofisher, Hemel Hempstead, UK) and processed using FreeStyle 1.8 (Thermofisher, Hemel Hempstead, UK) and TraceFinder 5.1 (Thermofisher, Hemel Hempstead, UK). 

## Figures and Tables

**Table 1:** Toxin concentrations quantified in clinical samples from dog using HILIC-MS/MS and LC-HRMS methods.

Sample	HILIC-MS/MS		LC-HRMS	
	ATX	dhATX	ATX	dhATX
Urine (ng/mL)	599	5,495	677	8165
Clot (ng/g)	nd	<LOQ	nd	nd
Stomach contents (ng/g)	1044	21,008	891	20,637
Blood (ng/mL)	nd	<LOQ	nd	nd

ATX = anatoxin-a; dhATX – dihydro-anatoxin-A; nd = not detected; <LOQ = below limit of quantitation. Note, dhATX quantified using ATX external calibration standard.

**Table 2:** Summary of LC-HRMS results detailing theoretical and measured *m*/*z* and mass error for anatoxin-a and dihydro anatoxin obtained in urine and stomach extracts.

Sample	Compound	Formula	Theoretical *m*/*z*	Measured *m*/*z*	Mass Error (ppm)
Urine	ATX	C_10_H_15_NO	166.1226	166.1227	0.602
Stomach	ATX	C_10_H_15_NO	166.1226	166.1227	0.602
Urine	dhATX	C_10_H_17_NO	168.1383	168.1381	−1.189
Stomach	dhATX	C_10_H_15_NO	168.1383	168.1381	−1.189

**Table 3:** Summary of LC-HRMS results detailing theoretical and measured *m*/*z*, mass error and retention time for minor anatoxin analogues obtained in stomach extract. Note: ND denotes not detected.

Compound	Formula	Theoretical *m*/*z*	Measured *m*/*z*	Mass Error (ppm)	RT (min)
HTX	C_11_H_17_NO	180.1383			ND
dhHTX	C_11_H_19_NO	182.1539			ND
4-keto-ATX	C_10_H_13_NO_2_	180.1019	180.1017	−1.11	5.54
epoxy ATX	C_10_H_15_NO_2_	182.1176	182.1175	−0.55	2.14
4-keto-HTX	C_11_H_15_NO_2_	194.1176	194.1175	−0.52	4.38
epoxy HTX	C_10_H_17_NO_2_	196.1332	196.1332	0	4.41, 6.22

**Table 4:** Summary of ATX detection and concentrations quantified in fatal canine cases.

Sample from Dog Stomach Contents		Environmental Samples	Species of Cyanobacteria and Location	Reference
ATX	dhATX	ATX		
1.04 µg/g	21.0 µg/g	na	na	This study
5980 µg/g ^a^	na	585 µg/L (water)	*Anabena* sp, Platt River, Nebraska	[56]
36,000 µg/g	4437 µg/kg	25 µg/L (ATX); 2118 µg/L (dhATX)	*Phormidium*, farm pond at Kaikoura, South Island, New Zealand	[31]
0.025 µg/g	0.56–1.18 µg/g	24.4–320 µg/L (mat from shoreline) 241–453 µg/L (floating mat)	*Tychonema* sp., Mandichosee, a reservoir of River Lech, south Germany	[15]
9.5 µg/g dw	NQ	272 µg/g (mat, dw)	*Phormidium* sp., Lake Ijmeer, Netherlands	[57]
5.7–8700 µg/kg	na	943–1870 µg/L (aqueous fraction of clumps of water moss, *Fontinalis antipyretica*)	*Tychonema* sp., Lake Tegel, Berlin, Germany	[58]
600 µg/g (liver)	na	8 mg/g (biofilm extract at shoreline)	*Phormidium favosum*, La Loue River, eastern France	[22]
NQ	HTX and dhATX (NQ)	9.4–27 µg/kg (ATX-a); 51–4400 µg/kg (HTX) (mat, ww)	*Phormidium* (probably *P. autumnale*), Hutt River, North Island, New Zealand	[32]
NQ	na	NQ (pond water)	Pond on a farm in Ontario, Canada	[59,60]
NQ	na	NQ (water)	Eel river, California, US	[60]
NQ (bile and urine)	na	NQ (water)	*Phormidium* sp., bucket of water from man-made pond, near Reno, Nevada	[61]
NQ	na	NQ (bacterial bloom)	*Oscillatoria* sp, Loch Insh, Scotland	[36]

^a^ also 0.3 mg/g MC-LR; na = not analysed for; NQ = confirmed but not quantified; dw = dry weight; ww = wet weight.

**Table 5:** Summary of mass spectral conditions utilised for determination of each cyanotoxin using LC-MS/MS methods.

Method	Toxin	SRM/SIM Transitions	Collision Energy (eV)
1-LC-MS/MS	MC-RR	519.9 > 134.9; 126.9	40; 60
	L-Nod	692.0 > 135.0; 107.0	60; 80
	Nod-R	825.5 > 135.1; 103.1	60; 100
	MC-LA	910.1 > 135.1; 106.9	65; 80
	[Asp3]-MC-LR	981.5 > 134.9; 106.9	60; 60
	MC-LF	986.5 > 213.0; 135.0	60; 65
	MC-LR	995.6 > 135.0; 127.0	65; 80
	MC-LY	1002.5 > 135.0; 106.9	75; 80
	MC-HilR	1009.7 > 134.9; 126.9	40; 60
	MC-LW	1025.5 > 134.9; 126.8	65; 90
	MC-YR	1045.6 > 135.0; 126.9	75; 80
	MC-HtyR	1059.6 > 134.9; 106.9	75; 80
	MC-WR	1068.6 > 134.9; 106.9	65; 80
	ATX	166.1 > 149.1; 131.1	20; 25
	CYN	416.1 > 336.2, 194.1	30; 45
	STX	300.1 > 204.1; 138.0	26; 30
2–HILIC-MS/MS	ATX	166.1 > 43.0; 91.0; 149.1	18; 18
	dhATX	168.0 > 56.0; 43.0; 93.0; 133.0	18; 18
	HTX	180.1 > 163.1; 145.1	18; 18
	dhHTX	182.0 > 164.1; 147.0	18; 18
	STX	300.1 > 204.1; 138.0	26; 30
	CYN	416.1 > 336.2, 194.1	30; 45

## Data Availability

Not applicable.

## Supplementary Materials

The following supporting information can be downloaded at: https://www.mdpi.com/article/10.3390/toxins14110804/s1, Figure S1: Product ion spectra of minor anatoxin analogues.
