# Supplementary material for: Confirmation Using Triple Quadrupole and High-Resolution Mass Spectrometry of a Fatal Canine Neurotoxicosis following Exposure to Anatoxins at an Inland Reservoir
Source: Toxins (Basel). 2022 Nov 18;14(11):804. doi: 10.3390/toxins14110804 (PMC9696769; doi:10.3390/toxins14110804)

Supplementary File

Confirmation Using Triple Quadrupole and High-Resolution Mass Spectrometry of a Fatal Canine Neurotoxicosis following Exposure to Anatoxins at an Inland Reservoir

Andrew D. Turner *, Florence R. I. Turner, Martha White, David Hartnell, Claire G. Crompton, Nicola Bates, Jan Egginton, Liz Branscombe, Adam M. Lewis and Benjamin H. Maskrey

* Correspondence: author: andrew.turner@cefas.co.uk; Tel.: +44-(0)1305-206636

Supplementary Figure S1. Product ion spectra of minor anatoxin analogues determined by LC-HRMS analysis

(A) 4-keto-ATX –*m/z* 180.1019. 5.53 mins

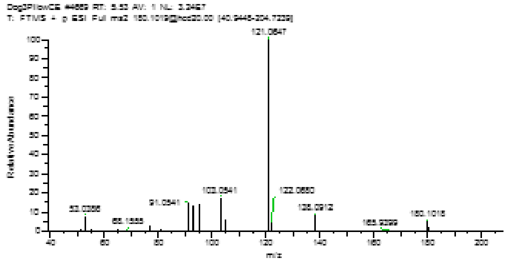

(B) epoxy ATX – *m/z* 182.1176. 2.20 mins

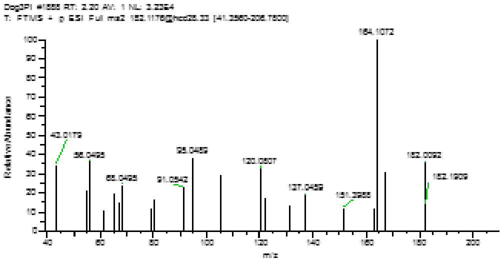

(C) 4-keto-HTX – *m/z* 194.1176

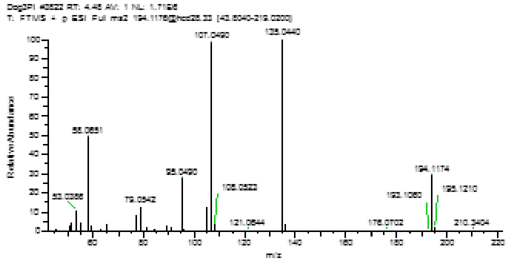

(D) epoxy-HTX – *m/z* 196.1332. 6.22 mins

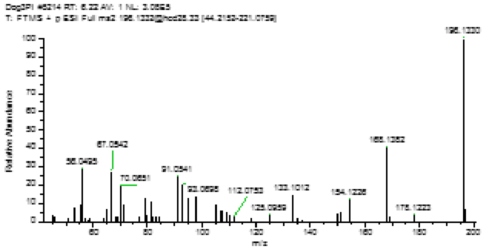

Supplement: Supplementary file 1 [file toxins-14-00804-s001.zip › toxins-1980986-supplementary.pdf]
